# Supplementary material for: The Evolution and Characterization of the RNA Interference Pathways in Lophotrochozoa
Source: Genome Biol Evol. 2024 May 7;16(5):evae098. doi: 10.1093/gbe/evae098 (PMC11114477; doi:10.1093/gbe/evae098)
Supplement: evae098_Supplementary_Data [file evae098_supplementary_data.zip › Supplementary_materials.zip!/selected_miRNAs/NoGe_selected_miRNAs/mirdeep_pdf_files/NMRB01000494.1_Notospermus_geniculatus_strain_Ushimado_scaffold494,_whole_genome_shotgun_sequence_14538.pdf]

Provisional ID	:	NMRB01000494.1_Notospermus_geniculatus_strain_Ushimado_scaffold494,_whole_genome_shotgun_sequence_14538
Score total	:	82762.4
Score for star read(s)	:	3.9
Score for read counts	:	82754.4
Score for mfe	:	1.1
Score for randfold	:	0
Score for cons. seed	:	3
Total read count	:	162331
Mature read count	:	162183
Loop read count	:	0
Star read count	:	148

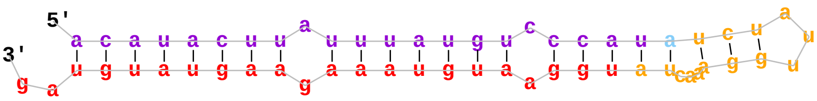

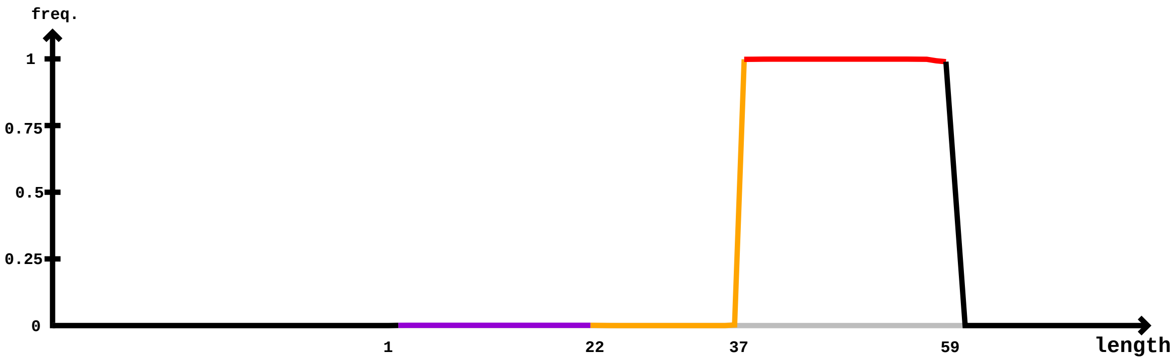

Star **Mature**

5'- cccaaaggaagcagaucacucacgugugagggucacauacuuauuuaugucccauaucuauuggaaacuauggaauguaaagaaguauguagcccuuccauguauauucaac -3'	obs			
cccaaaggaagcagaucacucacgugugagggucacauacuuauuuaugucccauaucuauuggaaacuauggaauguaaagaaguauguagcccuuccauguauauucaac	exp			
.....................(((((.((((((.((((((((.(((((((.((((((((...)))...))))).))))))).)))))))).)))))).)))))).........		reads	mm	sample
...................................cauacuuauuuaugucccaua..		1	0	Mm1
....auggaauguaaagaaguaugua.....................		6	0	Mm1
....Cuggaauguaaagaaguauguag....................		1	1	Mm1
....auggaauguaaagaaguauguaU....................		8	1	Mm1
....uggaauguaaagaaguau........................		4	0	Mm1
....uggaauguaaagaaguaug.......................		20	0	Mm1
....uggaauguaaagaaguaCg.......................		1	1	Mm1
....uggaauguaaCgaaguaugu......................		2	1	Mm1
....uggaauguaaagaaguCugu......................		1	1	Mm1
....uggaauguaaagaaguaugu......................		106	0	Mm1
....uggaauguaaagaaguaAgu......................		1	1	Mm1
....Aggaauguaaagaaguaugua.....................		1	1	Mm1
....uggaauguaaUgaaguaugua.....................		1	1	Mm1
....uggaauguaaagaaguaugua.....................		73	0	Mm1
....uggaauguaaagaaguUugua.....................		3	1	Mm1
....uggaauguaaagaaguaCguag....................		17	1	Mm1
....uggaauguaaagaaguauguCg....................		1	1	Mm1
....uggaauguaaagaaguCuguag....................		7	1	Mm1
....uggaaGguaaagaaguauguag....................		1	1	Mm1
....Aggaauguaaagaaguauguag....................		2	1	Mm1
....uggaauguUaagaaguauguag....................		1	1	Mm1
....uggaauguaaagaaguauguaA....................		5	1	Mm1
....uggaauUuaaagaaguauguag....................		1	1	Mm1
....uggaauguaaagaaguaAguag....................		12	1	Mm1
....uggaauguaGagaaguauguag....................		9	1	Mm1
....uggaauguaaagaaguaGguag....................		11	1	Mm1
....uggaauguaaagaGguauguag....................		1	1	Mm1
....uggaauguaaagaaguauguUg....................		1	1	Mm1
....uAgaauguaaagaaguauguag....................		3	1	Mm1
....uggaauguaaagaaguUuguag....................		3	1	Mm1
....uggaauguaaagaaguGuguag....................		7	1	Mm1
....uggaaCguaaagaaguauguag....................		1	1	Mm1
....uggaauguaaagaagAauguag....................		1	1	Mm1

```
                                  Star                                 Mature
cccaaaggaagcagaucacucacgugugaggguccacauacuuauuuaugucccauaucuauuggaaacuauggaauguaaagaaguauguagcccuuccauguauauucaac
......................................................................uCgaauguaaagaaguauguag....................    1      1    Mm1
......................................................................uggaauguaaagaaUuauguag....................    2      1    Mm1
......................................................................uggaauguaaagaaguauguaU....................    14     1    Mm1
......................................................................uggaauguaaagCaguauguag....................    1      1    Mm1
......................................................................uggaauguaaagaaguaugCag....................    1      1    Mm1
......................................................................uggaauguaaUgaaguauguag....................    7      1    Mm1
......................................................................uggaGuguaaagaaguauguag....................    1      1    Mm1
......................................................................Cggaauguaaagaaguauguag....................    2      1    Mm1
......................................................................uggaauguaaagGaguauguag....................    1      1    Mm1
......................................................................Nggaauguaaagaaguauguag....................    1      1    Mm1
......................................................................uggaauguaaagaaguauguag....................    3088   0    Mm1
......................................................................uggaauguaaGgaaguauguag....................    5      1    Mm1
......................................................................uggUauguaaagaaguauguag....................    2      1    Mm1
......................................................................uggaauguaaagaaguauguagU...................    1040   1    Mm1
......................................................................uggaauguaaagaaguauguagG...................    1      1    Mm1
......................................................................uggaauguaaagaaguauguagA...................    66     1    Mm1
.......................................................................ggaauguaaagaaguauguag....................    3      0    Mm1
.......................................................................ggaauguaaagaaguauguagU...................    1      1    Mm1
........................................................................gaauguaaagaaguauguagU...................    3      1    Mm1

..................................acauacuuauuuaugucccau.........................................................    2      0    Mm2
..................................acauacuuauuuaugucccaua........................................................    5      0    Mm2
........................................................ucuauuggaaacuauggaaugua.................................    1      0    Mm2
.....................................................................auggaauguaaagaaguaugu......................    2      0    Mm2
.....................................................................auggaauguaaagaaguaugua.....................    5      0    Mm2
.....................................................................auggaauguaaagaaguauguaU....................    1      1    Mm2
.....................................................................auggaauguaGagaaguauguag....................    3      1    Mm2
.....................................................................auggaauguaaagaaguauguag....................    15     0    Mm2
.....................................................................auggaauguaaagaaguGuguag....................    1      1    Mm2
.....................................................................Cuggaauguaaagaaguauguag....................    2      1    Mm2
......................................................................uggaauguaaagaaguau........................    2      0    Mm2
......................................................................uggaauguaaagaaguaug.......................    1      0    Mm2
......................................................................uggaauguaaagaaguaCgu......................    1      1    Mm2
......................................................................uggaauguaaagaaguaugu......................    53     0    Mm2
......................................................................uggaauguaaagaaguauguG.....................    2      1    Mm2
......................................................................uggaauguaaagaaguauguU.....................    2      1    Mm2
......................................................................uggaauguaaagaaguaugua.....................    24     0    Mm2
......................................................................uAgaauguaaagaaguaugua.....................    1      1    Mm2
......................................................................uggaauguaaagaaguaAgua.....................    1      1    Mm2
......................................................................uggaauguaaagaaguaGguag....................    61     1    Mm2
......................................................................uggaauguaaagaaguauguA.....................    7      1    Mm2
......................................................................uggaauguaaagaaCuauguag....................    1      1    Mm2
......................................................................uggaauguUaagaaguauguag....................    3      1    Mm2
......................................................................uggaauguaaagaaguaCguag....................    76     1    Mm2
......................................................................ugAaauguaaagaaguauguag....................    2      1    Mm2
......................................................................uggaauguaaagaaguaugGag....................    1      1    Mm2
......................................................................uggaauguaaagaaAuauguag....................    1      1    Mm2
......................................................................uggaCuguaaagaaguauguag....................    1      1    Mm2
......................................................................uggaauguaaagaaguauguaU....................    12     1    Mm2
......................................................................uggaaugGaaagaaguauguag....................    2      1    Mm2
......................................................................Cggaauguaaagaaguauguag....................    4      1    Mm2
......................................................................ugUaauguaaagaaguauguag....................    3      1    Mm2
......................................................................uggaauguaaagGaguauguag....................    4      1    Mm2
......................................................................uggaauguaaagaaguauUuag....................    1      1    Mm2
......................................................................Gggaauguaaagaaguauguag....................    2      1    Mm2
......................................................................uggaauguaaagaaguauguag....................    13859  0    Mm2
......................................................................uggaauguaaagaaguaugAag....................    1      1    Mm2
......................................................................Nggaauguaaagaaguauguag....................    7      1    Mm2
......................................................................uggUauguaaagaaguauguag....................    2      1    Mm2
......................................................................uggaauguaaagaaguauguGg....................    20     1    Mm2
......................................................................uggaaGguaaagaaguauguag....................    2      1    Mm2
......................................................................uggaauguaaagaaguaugCag....................    2      1    Mm2
......................................................................uggaGuguaaagaaguauguag....................    5      1    Mm2
......................................................................uggaauguaaagaaguCuguag....................    32     1    Mm2
......................................................................uggCauguaaagaaguauguag....................    2      1    Mm2
......................................................................uggaauguaaagaagAauguag....................    1      1    Mm2
......................................................................uggaauguaaagaagCauguag....................    2      1    Mm2
......................................................................uggaauUuaaagaaguauguag....................    1      1    Mm2
......................................................................uggaaNguaaagaaguauguag....................    2      1    Mm2
......................................................................uggaauguaGagaaguauguag....................    3      1    Mm2
```

```
                                 Star                              Mature
cccaaaggaagcagaucacucacgugugagggucacauacuuauuuaugucccauaucuauuggaaacuauggaauguaaagaaguauguagcccuuccauguauauucaac
......................................................................uggaauguCaagaaguauguag....................     1      1     Mm2
......................................................................uggaauguaaagaaguauguUg....................     1      1     Mm2
......................................................................uggaauguaaaUaaguauguag....................     1      1     Mm2
......................................................................uggaauguaaGgaaguauguag....................     22     1     Mm2
......................................................................uUgaauguaaagaaguauguag....................     4      1     Mm2
......................................................................uggaUuguaaagaaguauguag....................     1      1     Mm2
......................................................................uggaauguaaagaCguauguag....................     1      1     Mm2
......................................................................uggaauguaaagaaguUuguag....................     24     1     Mm2
......................................................................uggaauguaaagaGguauguag....................     2      1     Mm2
......................................................................uggaaCguaaagaaguauguag....................     2      1     Mm2
......................................................................uggaauAuaaagaaguauguag....................     1      1     Mm2
......................................................................uggaauguaaagaUguauguag....................     1      1     Mm2
......................................................................uggaauguaUagaaguauguag....................     1      1     Mm2
......................................................................uggaaugAaaagaaguauguag....................     4      1     Mm2
......................................................................uAgaauguaaagaaguauguag....................     6      1     Mm2
......................................................................uggaauguaaagaaguGuguag....................     15     1     Mm2
......................................................................uggaauNuaaagaaguauguag....................     1      1     Mm2
......................................................................uggaauguaaagaaUuauguag....................     2      1     Mm2
......................................................................Aggaauguaaagaaguauguag....................     5      1     Mm2
......................................................................uggaauguaaagaaguauguCg....................     1      1     Mm2
......................................................................uggaauguaaUgaaguauguag....................     17     1     Mm2
......................................................................uggaauguaaagaaguaAguag....................     62     1     Mm2
......................................................................uggaauguaCagaaguauguag....................     1      1     Mm2
......................................................................uCgaauguaaagaaguauguag....................     3      1     Mm2
......................................................................uggaaugCaaagaaguauguag....................     1      1     Mm2
......................................................................uggGauguaaagaaguauguag....................     6      1     Mm2
......................................................................uggaauguaaagUaguauguag....................     5      1     Mm2
......................................................................uggaauguaaagCaguauguag....................     1      1     Mm2
......................................................................uggaauguaaaAaaguauguag....................     1      1     Mm2
......................................................................uggaauguGaagaaguauguag....................     3      1     Mm2
......................................................................uggaauguaaCgaaguauguag....................     12     1     Mm2
......................................................................uggaauguaaagaaguauguagc...................     2      0     Mm2
......................................................................uggaauguaaagaaguauguagU...................     3513   1     Mm2
......................................................................uggaauguaaagaaguauguagG...................     3      1     Mm2
......................................................................uggaauguaaagaaguauguagA...................     94     1     Mm2
.......................................................................ggaauguaaagaaguaGguag....................     1      1     Mm2
.......................................................................Ugaauguaaagaaguauguag....................     2      1     Mm2
.......................................................................ggaauguaaagaaguauguag....................     3      0     Mm2
.......................................................................ggaauguaaagaaguauguagU...................     2      1     Mm2
........................................................................gaauguaaagaaguauguagU...................     2      1     Mm2
.........................................................................aauguaaagaaguauguagA...................     1      1     Mm2

................................ucacauacuuauuuaugucccа..........................................................     4      0     Fm1
.................................cacauacuuauuuaugucccau.........................................................     2      0     Fm1
..................................acauacuuauuuauguccc...........................................................     6      0     Fm1
..................................acauacuuauuuaugucAcau.........................................................     1      1     Fm1
..................................acauacuuauuuaugucccau.........................................................     32     0     Fm1
..................................acauacuuauuuaugucccaua........................................................     26     0     Fm1
..................................acauacuuauuuaugucccauau.......................................................     5      0     Fm1
...................................cauacuuauuuaugucccau.........................................................     8      0     Fm1
...................................cauacuuauuuaugucccaua........................................................     17     0     Fm1
...................................cauacuuauuuaugucccauaC.......................................................     3      1     Fm1
...........................................................uauuggaaacuauggaauguaaagaaguauguag....................     2      0     Fm1
.....................................................................auggaauguaaagaaguaugu......................     10     0     Fm1
.....................................................................auggaauguaaagaaguaugua.....................     9      0     Fm1
.....................................................................auggaauguaaagaaguauguag....................     28     0     Fm1
.....................................................................auggaauguaaagaaguauguaU....................     3      1     Fm1
.....................................................................auggaauguaGagaaguauguag....................     5      1     Fm1
.....................................................................Cuggaauguaaagaaguauguag....................     9      1     Fm1
.....................................................................auggaauguaaagaaguauguagU...................     4      1     Fm1
......................................................................uggaauguaaagaaguau........................     3      0     Fm1
......................................................................uggaauguaaagaaguaug.......................     2      0     Fm1
......................................................................uggaauguaaUgaaguaugu......................     1      1     Fm1
......................................................................uggaauguaaagaaguUugu......................     1      1     Fm1
......................................................................uggaauguaaagaaguaugG......................     1      1     Fm1
......................................................................uggaauguaaagaaguaCgu......................     1      1     Fm1
......................................................................uggaauguaaGgaaguaugu......................     1      1     Fm1
......................................................................uggaauguaaCgaaguaugu......................     1      1     Fm1
......................................................................uggaauguaaagaaguaugu......................     194    0     Fm1
......................................................................uggaauguaaagaaguaGgu......................     1      1     Fm1
```

Star Mature

```
cccaaaggaagcagaucacucacgugugagggucacauacuuauuuaugucccauaucuauuggaaacuauggaauguaaagaaguauguagcccuuccauguauauucaac
......................................................................uggaauguaaagaaguaAgu......................	1	1	Fm1
......................................................................uggaauguaaagaaguauguU.....................	2	1	Fm1
......................................................................uggaauguaaagaaguaGgua.....................	1	1	Fm1
......................................................................uggaauguaaagaagAaugua.....................	1	1	Fm1
......................................................................uggaauguaaagaaguaugua.....................	80	0	Fm1
......................................................................uggaauguaaagaaguauguG.....................	1	1	Fm1
......................................................................uggaauguaaagaaguauguaU....................	22	1	Fm1
......................................................................uggCauguaaagaaguauguag....................	14	1	Fm1
......................................................................uggaauguaGagaaguauguag....................	8	1	Fm1
......................................................................uggaauguaaagaagCauguag....................	5	1	Fm1
......................................................................uggaauguaaagaGguauguag....................	17	1	Fm1
......................................................................uggaauguaaagaaguauguag....................	44471	0	Fm1
......................................................................uggaCuguaaagaaguauguag....................	8	1	Fm1
......................................................................uggaauguaaagaaguUuguag....................	52	1	Fm1
......................................................................Aggaauguaaagaaguauguag....................	7	1	Fm1
......................................................................uCgaauguaaagaaguauguag....................	7	1	Fm1
......................................................................ugUaauguaaagaaguauguag....................	9	1	Fm1
......................................................................uggaaCguaaagaaguauguag....................	7	1	Fm1
......................................................................uggaauguaaagaaguaCguag....................	302	1	Fm1
......................................................................uggaauguaaagaagAauguag....................	1	1	Fm1
......................................................................uggaGuguaaagaaguauguag....................	12	1	Fm1
......................................................................uggaauguaaagaUguauguag....................	10	1	Fm1
......................................................................uggaaAguaaagaaguauguag....................	1	1	Fm1
......................................................................uggUauguaaagaaguauguag....................	6	1	Fm1
......................................................................uggaauguaaagaaguaugCag....................	5	1	Fm1
......................................................................uggaUuguaaagaaguauguag....................	3	1	Fm1
......................................................................uggaauguaaagCaguauguag....................	5	1	Fm1
......................................................................uggaauguaaagaaguGuguag....................	59	1	Fm1
......................................................................uggaauguaaCgaaguauguag....................	35	1	Fm1
......................................................................uggaauguaaGgaaguauguag....................	64	1	Fm1
......................................................................uggaauguaaagaaguCuguag....................	111	1	Fm1
......................................................................uggaauguaaagaaguauguaA....................	6	1	Fm1
......................................................................uggaauguaaagaaguauCuag....................	2	1	Fm1
......................................................................uggaauguaCagaaguauguag....................	2	1	Fm1
......................................................................uggGauguaaagaaguauguag....................	12	1	Fm1
......................................................................uggaauguaUagaaguauguag....................	4	1	Fm1
......................................................................ugCaauguaaagaaguauguag....................	5	1	Fm1
......................................................................uggaaGguaaagaaguauguag....................	1	1	Fm1
......................................................................uggaauguaaagGaguauguag....................	8	1	Fm1
......................................................................uggaauguaaagaaguauguUg....................	4	1	Fm1
......................................................................uggaauguaaagaaguaugGag....................	7	1	Fm1
......................................................................uggaauguaaagaaguaugAag....................	6	1	Fm1
......................................................................uggaauguGaagaaguauguag....................	5	1	Fm1
......................................................................uggaauguaaagaaguauguCg....................	1	1	Fm1
......................................................................uggaauguaaagaCguauguag....................	4	1	Fm1
......................................................................uggaauguaaagaagGauguag....................	2	1	Fm1
......................................................................uggaauCuaaagaaguauguag....................	4	1	Fm1
......................................................................uggaauguaaagaaguauAuag....................	4	1	Fm1
......................................................................Gggaauguaaagaaguauguag....................	1	1	Fm1
......................................................................Cggaauguaaagaaguauguag....................	15	1	Fm1
......................................................................uggaauguaaagaaguauguGg....................	62	1	Fm1
......................................................................uggaaugCaaagaaguauguag....................	3	1	Fm1
......................................................................uggaauguUaagaaguauguag....................	17	1	Fm1
......................................................................uggaauguaaagaaCuauguag....................	1	1	Fm1
......................................................................uggaauguCaagaaguauguag....................	4	1	Fm1
......................................................................uggaauguaaagaaUuauguag....................	5	1	Fm1
......................................................................uggaauguaaaUaaguauguag....................	4	1	Fm1
......................................................................uggaauguaaagaaguaAguag....................	231	1	Fm1
......................................................................uAgaauguaaagaaguauguag....................	23	1	Fm1
......................................................................uggaaugGaaagaaguauguag....................	1	1	Fm1
......................................................................Nggaauguaaagaaguauguag....................	30	1	Fm1
......................................................................uggaauguaaagaaguaGguag....................	204	1	Fm1
......................................................................ugAaauguaaagaaguauguag....................	4	1	Fm1
......................................................................uggaaNguaaagaaguauguag....................	7	1	Fm1
......................................................................uggaauguaaagUaguauguag....................	34	1	Fm1
......................................................................uggaauguaaUgaaguauguag....................	47	1	Fm1
......................................................................uggaauAuaaagaaguauguag....................	8	1	Fm1
......................................................................uggaauguaaagaaguauUuag....................	1	1	Fm1
......................................................................uggaauUuaaagaaguauguag....................	4	1	Fm1
......................................................................uggaauguaaagaaAuauguag....................	2	1	Fm1
```

Star Mature

cccaaaggaagcagaucacucacgugugagggucacauacuuauuuaugucccauaucuauuggaaacuauggaauguaaagaaguauguagcccuuccauguauauucaac			
....uggaaugAaaagaaguauguag....................	5	1	Fm1
....uUgaauguaaagaaguauguag....................	7	1	Fm1
....uggaauguaaagaaguauguaC....................	10	1	Fm1
....uggaauguaaagaaguauguagG...................	1	1	Fm1
....uggaauguaaagaaguauguagA...................	144	1	Fm1
....uggaauguaaagaaguauguagU...................	3239	1	Fm1
....ggaauguaaagaaguaugu......................	2	0	Fm1
....ggaauguaaagaaguauguag....................	3	0	Fm1
....Ugaauguaaagaaguauguag....................	1	1	Fm1
....ggaauguaaagaaguauguagU...................	1	1	Fm1
....gaauguaaagaaguauguag....................	3	0	Fm1
....gaauguaaagaaguauguagU...................	1	1	Fm1
....aauguaaagaaguaugGag....................	1	1	Fm1
..................................acauacuuauGuaugucccau...	1	1	Fm2
..................................acauacuuauuuaugucccau...	3	0	Fm2
..................................acauacuuauuuaugucccauaA...	11	1	Fm2
....auggaauguaaagaagua.........................	1	0	Fm2
....auggaauguaaagaaguaugu......................	2	0	Fm2
....auggaauguaaagaaguaugG......................	1	1	Fm2
....auggaauguaaagaagCauguag....................	1	1	Fm2
....auggaauguaaagaaguauguag....................	8	0	Fm2
....auggaauguaaagaaguauguagU...................	12	1	Fm2
....uggaauguaaagaaguau........................	3	0	Fm2
....uggaauguaaagaaguUu........................	3	1	Fm2
....uggaauguaaagaaguCug.......................	1	1	Fm2
....uggaauguaaagaaguaug.......................	10	0	Fm2
....uggaauguaaagaaguaugu......................	197	0	Fm2
....uggaauguaaagaaguCugu......................	1	1	Fm2
....uggaauguaaagaaguaCgu......................	1	1	Fm2
....uggaauguGaagaaguaugu......................	1	1	Fm2
....uggaauguaaagaaguaGgu......................	1	1	Fm2
....uggaauguaaagaaguaAgu......................	1	1	Fm2
....uggaauguaaagaaguaGgua.....................	1	1	Fm2
....uggaauguaaagaaguauguG.....................	2	1	Fm2
....uggaauguaaagaaguaugua.....................	148	0	Fm2
....uggaauguaaagaaguaCgua.....................	1	1	Fm2
....uggaauguaGagaaguaugua.....................	4	1	Fm2
....uggaauguaaagaaguaAgua.....................	1	1	Fm2
....uggaauguaaagaaguUugua.....................	1	1	Fm2
....Gggaauguaaagaaguauguag....................	1	1	Fm2
....uggaaugCaaagaaguauguag....................	4	1	Fm2
....uggaauguaaagaaguauguGg....................	54	1	Fm2
....uggaaAguaaagaaguauguag....................	1	1	Fm2
....uCgaauguaaagaaguauguag....................	2	1	Fm2
....uggCauguaaagaaguauguag....................	14	1	Fm2
....uggaaugGaaagaaguauguag....................	1	1	Fm2
....uggaaCguaaagaaguauguag....................	3	1	Fm2
....ugCaauguaaagaaguauguag....................	1	1	Fm2
....uggaauguaaagaaguauguCg....................	3	1	Fm2
....uggaauguaCagaaguauguag....................	6	1	Fm2
....uggaauguaaagaUguauguag....................	9	1	Fm2
....uggaauguaGagaaguauguag....................	23	1	Fm2
....uggaauguaaagaagCauguag....................	4	1	Fm2
....uggaauguaaagCaguauguag....................	1	1	Fm2
....uggaauguCaagaaguauguag....................	2	1	Fm2
....uggaauguaaagaaguaGguag....................	175	1	Fm2
....uggaCuguaaagaaguauguag....................	2	1	Fm2
....uggaGuguaaagaaguauguag....................	5	1	Fm2
....uggaauguaaagaagGauguag....................	1	1	Fm2
....uggaauguaaagGaguauguag....................	4	1	Fm2
....uggaauguaUagaaguauguag....................	6	1	Fm2
....Aggaauguaaagaaguauguag....................	13	1	Fm2
....ugAaauguaaagaaguauguag....................	3	1	Fm2
....uggaauguaaagaaguauguaA....................	28	1	Fm2
....uggaauguaaagUaguauguag....................	9	1	Fm2
....uggaauguaaagaaUuauguag....................	4	1	Fm2
....uggaaNguaaagaaguauguag....................	6	1	Fm2
....uggaauguaaCgaaguauguag....................	28	1	Fm2
....uggaauguaaaUaaguauguag....................	2	1	Fm2

```
                                  Star                              Mature
cccaaaggaagcagaucacucacgugugagggucacauacuuauuuaugucccauaucuauuggaaacuauggaauguaaagaaguauguagcccuuccauguauauucaac
......................................................................uggaauguaaagaaguaAguag....................   163     1    Fm2
......................................................................uggaauguaaagaaguauguaU....................   31      1    Fm2
......................................................................uggaauguaaagaaguaugGag....................   4       1    Fm2
......................................................................Cggaauguaaagaaguauguag....................   7       1    Fm2
......................................................................uggaauguaaagaaguaCguag....................   219     1    Fm2
......................................................................uUgaauguaaagaaguauguag....................   11      1    Fm2
......................................................................uggaauguaaagaaguaugAag....................   5       1    Fm2
......................................................................uggaauguaaagaaguCuguag....................   76      1    Fm2
......................................................................Nggaauguaaagaaguauguag....................   13      1    Fm2
......................................................................uggaauguaaagaaguauCuag....................   1       1    Fm2
......................................................................uggaauguUaagaaguauguag....................   13      1    Fm2
......................................................................uggaUuguaaagaaguauguag....................   2       1    Fm2
......................................................................uggaauguaaaCaaguauguag....................   2       1    Fm2
......................................................................uggaauguaaagaaguauguag....................   34009   0    Fm2
......................................................................uggaauUuaaagaaguauguag....................   3       1    Fm2
......................................................................uggaauguaaGgaaguauguag....................   43      1    Fm2
......................................................................uggaauguaaagaGguauguag....................   2       1    Fm2
......................................................................uggaauAuaaagaaguauguag....................   1       1    Fm2
......................................................................uggaauguaaagaaguauAuag....................   4       1    Fm2
......................................................................uggaaugAaaagaaguauguag....................   5       1    Fm2
......................................................................uggaauguaaagaaCuauguag....................   1       1    Fm2
......................................................................uggaauguaaagaaguaugCag....................   7       1    Fm2
......................................................................uggaaGguaaagaaguauguag....................   1       1    Fm2
......................................................................uggGauguaaagaaguauguag....................   12      1    Fm2
......................................................................uggaauguaaagaagAauguag....................   2       1    Fm2
......................................................................uggaauguaaagaaguauguaC....................   7       1    Fm2
......................................................................uggaauguGaagaaguauguag....................   5       1    Fm2
......................................................................uggaauguaaagaaguUuguag....................   39      1    Fm2
......................................................................uAgaauguaaagaaguauguag....................   21      1    Fm2
......................................................................ugUaauguaaagaaguauguag....................   9       1    Fm2
......................................................................uggUauguaaagaaguauguag....................   7       1    Fm2
......................................................................uggaauguaaagaaAuauguag....................   1       1    Fm2
......................................................................uggaauguaaagaaguGuguag....................   44      1    Fm2
......................................................................uggaauguaaaAaaguauguag....................   2       1    Fm2
......................................................................uggaauguaaUgaaguauguag....................   43      1    Fm2
......................................................................uggaauguaaagaaguauguagc...................   1       0    Fm2
......................................................................uggaauguaaagaaguauguagU...................   6574    1    Fm2
......................................................................uggaauguaaagaaguauguagG...................   12      1    Fm2
......................................................................uggaauguaaagaaguauguagA...................   201     1    Fm2
......................................................................uggaauguaaagaaguauguagcU..................   1       1    Fm2
.......................................................................ggaauguaaagaaguaugua.....................   1       0    Fm2
.......................................................................ggaauguaaagaaguauguag....................   1       0    Fm2
........................................................................gaauguaaagaaguauguag....................   5       0    Fm2
........................................................................gaauguaaagaaguauguagU...................   6       1    Fm2
.........................................................................aauguaaagaaguauguagA...................   4       1    Fm2
.........................................................................aauguaaagaaguauguagU...................   3       1    Fm2

..................................acauacuuauuuaugucccau.........................................................   1       0    Mm3
..................................acauacuuauuuaugucccauU........................................................   1       1    Mm3
..................................acauacuuauuuaugucccaua........................................................   1       0    Mm3
..................................acauacuuauuuaugucccauau.......................................................   1       0    Mm3
...................................cauacuuauuuaugucccaua........................................................   1       0    Mm3
.....................................................................auggaauguaaagaaguaugu......................   4       0    Mm3
.....................................................................auggaauguaGagaaguaugua.....................   1       1    Mm3
.....................................................................auggaauguaaagaaguauguU.....................   2       1    Mm3
.....................................................................auggaauguaaagaaguaugua.....................   1       0    Mm3
.....................................................................auggaauguaaagaaguauguag....................   11      0    Mm3
.....................................................................auggaauguaaagaaguauguaU....................   1       1    Mm3
.....................................................................auggaauguaGagaaguauguag....................   1       1    Mm3
.....................................................................auggaauguaUagaaguauguag....................   1       1    Mm3
.....................................................................Cuggaauguaaagaaguauguag....................   4       1    Mm3
.....................................................................auggaauguaaagaaguauguagU...................   1       1    Mm3
......................................................................uggaauguaaagaaguau........................   2       0    Mm3
......................................................................uggaauguaaagaaguaug.......................   6       0    Mm3
......................................................................uggaauguaaagaaguaugC......................   1       1    Mm3
......................................................................uggaauguaaagaaguaugu......................   48      0    Mm3
......................................................................uggaauguaaagaaguaugA......................   1       1    Mm3
......................................................................uggaauguaaagaaguauguU.....................   1       1    Mm3
......................................................................uggaauguaaagaaguaugua.....................   37      0    Mm3
......................................................................uggaauguaaagaaguaGgua.....................   1       1    Mm3
```

Star **Mature**

```
cccaaaggaagcagaucacucacgugugagggucacauacuuauuuaugucccauaucuauuggaaacuauggaauguaaagaaguauguagcccuuccauguauauucaac
......................................................................uggaauguaaagaaguauguGg....................   3      1   Mm3
......................................................................ugUaauguaaagaaguauguag....................   1      1   Mm3
......................................................................uggaauguaaCgaaguauguag....................   7      1   Mm3
......................................................................uggaauguaaagaaguauguaC....................   2      1   Mm3
......................................................................uggaauNuaaagaaguauguag....................   1      1   Mm3
......................................................................uggaauguaaagaaguauguag....................   3532   0   Mm3
......................................................................uggaauguaaagCaguauguag....................   1      1   Mm3
......................................................................ugCaauguaaagaaguauguag....................   1      1   Mm3
......................................................................uggaauguaaagaaguaugCag....................   2      1   Mm3
......................................................................uggaauguUaagaaguauguag....................   2      1   Mm3
......................................................................uggaauguaaagaaguauguaU....................   6      1   Mm3
......................................................................uggaauUuaaagaaguauguag....................   1      1   Mm3
......................................................................uggaauguaaUgaaguauguag....................   3      1   Mm3
......................................................................uggaauguaaagaCguauguag....................   1      1   Mm3
......................................................................uggaauguaaagaaguCuguag....................   12     1   Mm3
......................................................................Cggaauguaaagaaguauguag....................   1      1   Mm3
......................................................................uggaauguaaagGaguauguag....................   1      1   Mm3
......................................................................uggaauguaaagaaguauguaA....................   9      1   Mm3
......................................................................uggaauguaaagaaguaAguag....................   11     1   Mm3
......................................................................uggaauguaaagaaguaGguag....................   18     1   Mm3
......................................................................Nggaauguaaagaaguauguag....................   2      1   Mm3
......................................................................uggaauguaaagaaguaCguag....................   31     1   Mm3
......................................................................uggaauguaaagUaguauguag....................   3      1   Mm3
......................................................................uggaauguaaagaaguGuguag....................   6      1   Mm3
......................................................................uggaauguaaagaaguUuguag....................   4      1   Mm3
......................................................................uUgaauguaaagaaguauguag....................   1      1   Mm3
......................................................................uggaaCguaaagaaguauguag....................   1      1   Mm3
......................................................................uggaauguaUagaaguauguag....................   1      1   Mm3
......................................................................uggCauguaaagaaguauguag....................   4      1   Mm3
......................................................................uggaauguaCagaaguauguag....................   1      1   Mm3
......................................................................uggaauguaGagaaguauguag....................   1      1   Mm3
......................................................................uggaauguaaagaagAauguag....................   1      1   Mm3
......................................................................uggaauguaaGgaaguauguag....................   3      1   Mm3
......................................................................uggaauguaaagaaguauguagc...................   1      0   Mm3
......................................................................uggaauguaaagaaguauguagA...................   55     1   Mm3
......................................................................uggaauguaaagaaguauguagU...................   1258   1   Mm3
.......................................................................ggaauguaaagaaguauguag....................   1      0   Mm3
.......................................................................Ugaauguaaagaaguauguag....................   2      1   Mm3
........................................................................gaauguaaagaaguauguag....................   3      0   Mm3
........................................................................gaauguaaagaaguauguagA...................   1      1   Mm3
........................................................................gaauguaaagaaguauguagU...................   4      1   Mm3
..........................................................................auguaaagaaguauguagU...................   1      1   Mm3

..................................acauacuuauuuaugucccau.........................................................   8      0   Fm3
..................................acauacuuauuuaugucccaGa........................................................   1      1   Fm3
..................................acauacuuauuuaugucccaua........................................................   4      0   Fm3
..................................acauacuuauuuaugucccauau.......................................................   1      0   Fm3
..................................acauacuuauuuaugucccauaA.......................................................   2      1   Fm3
....................................................................uauggaauguaaagaaguaugu......................   1      0   Fm3
.....................................................................auggaauguaaagaaguau........................   1      0   Fm3
.....................................................................auggaauguaaagaagCaugu......................   1      1   Fm3
.....................................................................auggaauguaaagaaguaugC......................   1      1   Fm3
.....................................................................auggaauguaaagaaguaugu......................   40     0   Fm3
.....................................................................auggaauguaGagaaguaugu......................   5      1   Fm3
.....................................................................auggaauguaaagaGguaugua.....................   3      1   Fm3
.....................................................................auggaauguaaagaaguauguU.....................   2      1   Fm3
.....................................................................auggaauguaaagaaguaugua.....................   16     0   Fm3
.....................................................................auggaauguaaagaaguauguag....................   74     0   Fm3
.....................................................................auggaauguaGagaaguauguag....................   5      1   Fm3
.....................................................................auggaauguaaagaagCauguag....................   2      1   Fm3
.....................................................................auggaauguaaagaagAauguag....................   2      1   Fm3
.....................................................................Cuggaauguaaagaaguauguag....................   2      1   Fm3
.....................................................................auggaauguaaagaaguauguaU....................   11     1   Fm3
.....................................................................auggaauguaCagaaguauguag....................   1      1   Fm3
.....................................................................Guggaauguaaagaaguauguag....................   1      1   Fm3
.....................................................................auAgaauguaaagaaguauguag....................   1      1   Fm3
.....................................................................auggaauguaaagaaguauguagU...................   17     1   Fm3
......................................................................uggaauguaaagaaguau........................   11     0   Fm3
......................................................................uggaauguaaagaaguaA........................   9      1   Fm3
......................................................................uggaauguaaagaaguaG........................   1      1   Fm3
```

```
                                  Star                                 Mature
cccaaaggaagcagaucacucacgugugagggucacauacuuauuuaugucccauaucuauuggaaacuauggaauguaaagaaguauguagcccuuccauguauauucaac
......................................................................uggaauguaaagaaguaC........................   1       1    Fm3
......................................................................uggaauguaaagaaguaug.......................   8       0    Fm3
......................................................................uggaauguaaagaaguaugA......................   3       1    Fm3
......................................................................uggaauguaaagaaguaGgu......................   1       1    Fm3
......................................................................uggaauguaaagaaguaCgu......................   4       1    Fm3
......................................................................uggaauguaaagaaguaugu......................   224     0    Fm3
......................................................................uggaauguGaagaaguaugu......................   2       1    Fm3
......................................................................Nggaauguaaagaaguaugu......................   1       1    Fm3
......................................................................uggaauguaaagaaguaAgu......................   2       1    Fm3
......................................................................uggaauguaaagaaguauguG.....................   2       1    Fm3
......................................................................uggaauguaaagaaguaAgua.....................   1       1    Fm3
......................................................................uggaauguaaagaaguaGgua.....................   1       1    Fm3
......................................................................uggaauguaaagaaguaugua.....................   99      0    Fm3
......................................................................uggaauguaaagaaguaCgua.....................   2       1    Fm3
......................................................................uggaauguaaagaaguaugAa.....................   1       1    Fm3
......................................................................uggaauguaaagaaguauguU.....................   7       1    Fm3
......................................................................uggaauguaaagaaguUugua.....................   1       1    Fm3
......................................................................Aggaauguaaagaaguauguag....................   10      1    Fm3
......................................................................uggaCuguaaagaaguauguag....................   10      1    Fm3
......................................................................ugCaauguaaagaaguauguag....................   2       1    Fm3
......................................................................uggaauguaaagaaguaugAag....................   1       1    Fm3
......................................................................uAgaauguaaagaaguauguag....................   13      1    Fm3
......................................................................uggaauguUaagaaguauguag....................   14      1    Fm3
......................................................................uggaauguaaagaagCauguag....................   2       1    Fm3
......................................................................uggaauCuaaagaaguauguag....................   1       1    Fm3
......................................................................uggaauguaaaAaaguauguag....................   2       1    Fm3
......................................................................Cggaauguaaagaaguauguag....................   7       1    Fm3
......................................................................uggaauguGaagaaguauguag....................   7       1    Fm3
......................................................................uggaauguaaagaaguGuguag....................   39      1    Fm3
......................................................................uggaaGguaaagaaguauguag....................   2       1    Fm3
......................................................................uggaauguaaagaaguauguUg....................   4       1    Fm3
......................................................................Nggaauguaaagaaguauguag....................   14      1    Fm3
......................................................................uUgaauguaaagaaguauguag....................   19      1    Fm3
......................................................................uggaauguaaagaaguauguaC....................   2       1    Fm3
......................................................................uggaauguaaagaaguaAguag....................   159     1    Fm3
......................................................................uggaauguaaagaaUuauguag....................   4       1    Fm3
......................................................................uggaaNguaaagaaguauguag....................   7       1    Fm3
......................................................................uggaauguaGagaaguauguag....................   5       1    Fm3
......................................................................uggaauguaaagaaguUuguag....................   46      1    Fm3
......................................................................uggaauguaaaUaaguauguag....................   7       1    Fm3
......................................................................uggaauguaaUgaaguauguag....................   20      1    Fm3
......................................................................uggaaAguaaagaaguauguag....................   2       1    Fm3
......................................................................uggaauguaaagaUguauguag....................   5       1    Fm3
......................................................................ugUaauguaaagaaguauguag....................   13      1    Fm3
......................................................................uggaauguaaagaaguauguaU....................   55      1    Fm3
......................................................................uggaauguaaagaaguauUuag....................   2       1    Fm3
......................................................................uggaauguCaagaaguauguag....................   1       1    Fm3
......................................................................uggaauguaaagaaguaGguag....................   179     1    Fm3
......................................................................uggaauguaaagaaguauguag....................   32344   0    Fm3
......................................................................uggaauguaaagaaguaugCag....................   5       1    Fm3
......................................................................uggaaugAaaagaaguauguag....................   4       1    Fm3
......................................................................uggaauguaaagaGguauguag....................   19      1    Fm3
......................................................................uggaauguaaagaaguauguGg....................   15      1    Fm3
......................................................................uggaauguaCagaaguauguag....................   4       1    Fm3
......................................................................ugAaauguaaagaaguauguag....................   6       1    Fm3
......................................................................uggaauguaaCgaaguauguag....................   18      1    Fm3
......................................................................uggGauguaaagaaguauguag....................   11      1    Fm3
......................................................................uggaaugCaaagaaguauguag....................   7       1    Fm3
......................................................................uggaauguaaGgaaguauguag....................   33      1    Fm3
......................................................................uggaauNuaaagaaguauguag....................   4       1    Fm3
......................................................................uggaauguaaagaCguauguag....................   1       1    Fm3
......................................................................uggaauguaaagGaguauguag....................   4       1    Fm3
......................................................................Gggaauguaaagaaguauguag....................   1       1    Fm3
......................................................................uggaauguaaagaaguauguCg....................   1       1    Fm3
......................................................................uggaauguaaagaaguauguaA....................   34      1    Fm3
......................................................................uggaauguaUagaaguauguag....................   8       1    Fm3
......................................................................uggaauguaaagaaguCuguag....................   65      1    Fm3
......................................................................uggaUuguaaagaaguauguag....................   3       1    Fm3
......................................................................uggaauguaaagaagAauguag....................   2       1    Fm3
......................................................................uggaauguaaagaaAuauguag....................   1       1    Fm3
```

```
                                  Star                               Mature
cccaaaggaagcagaucacucacgugugagggucacauacuuauuuaugucccauaucuauuggaaacuauggaauguaaagaaguauguagcccuuccauguauauucaac
......................................................................uggaaCguaaagaaguauguag....................   3      1   Fm3
......................................................................uggCauguaaagaaguauguag....................   11     1   Fm3
......................................................................uggaGuguaaagaaguauguag....................   2      1   Fm3
......................................................................uCgaauguaaagaaguauguag....................   4      1   Fm3
......................................................................uggaauUuaaagaaguauguag....................   2      1   Fm3
......................................................................uggaauguaaagaaguauAuag....................   2      1   Fm3
......................................................................uggaauAuaaagaaguauguag....................   3      1   Fm3
......................................................................uggUauguaaagaaguauguag....................   6      1   Fm3
......................................................................uggaauguaaagUaguauguag....................   4      1   Fm3
......................................................................uggaauguaaagaaguaCguag....................   181    1   Fm3
......................................................................uggaauguaaagaaguauguagU...................   7743   1   Fm3
......................................................................uggaauguaaagaaguauguagA...................   352    1   Fm3
.......................................................................ggaauguaaagaaguauguaA....................   1      1   Fm3
.......................................................................ggaauguaaagaaguauguag....................   10     0   Fm3
.......................................................................Ugaauguaaagaaguauguag....................   9      1   Fm3
.......................................................................ggaauguaaagaaguauguagU...................   4      1   Fm3
........................................................................gaauguaaagaaguauguag....................   9      0   Fm3
........................................................................gaauguaaagaaguauguagA...................   13     1   Fm3
........................................................................gaauguaaagaaguauguagU...................   17     1   Fm3
.........................................................................aauguaaagaaguauguag....................   2      0   Fm3
.........................................................................aauguaaagaaguauguagU...................   2      1   Fm3
...........................................................................uguaaagaaguauguagU...................   2      1   Fm3
```
